# Efficacy of the Online Interactive Podcast Program “Living With Type 1 Diabetes to Grown-Up”: A Two-Arm Randomized Controlled Trial Protocol

**DOI:** 10.1155/jdr/4866975

**Published:** 2025-09-13

**Authors:** Yueh-Tao Chiang, Fu-Sung Lo, Hsing-Yi Yu, Chi-Wen Chen, Philip Moons

**Affiliations:** ^1^Department of Nursing, College of Medicine, Chang Gung University, Taoyuan, Taiwan; ^2^Division of Pediatric Endocrinology & Genetics Department of Pediatrics, Chang-Gung Memorial Hospital, Taoyuan, Taiwan; ^3^College of Medicine, Chang Gung University, Taoyuan, Taiwan; ^4^Department of Nursing, New Taipei Municipal Tu Cheng Hospital, New Taipei, Taiwan; ^5^College of Nursing, National Yang Ming Chiao Tung University, Taipei, Taiwan; ^6^Department of Public Health and Primary Care, KU Leuven, Leuven, Belgium; ^7^Centre for Person-Centred Care (GPCC), Sahlgrenska Academy, University of Gothenburg, Gothenburg, Sweden; ^8^Department of Paediatrics and Child Health, University of Cape Town, Cape Town, South Africa

**Keywords:** clinical efficacy, diabetes, health promotion, podcast, research in practice, self-care, technology

## Abstract

**Aim:** The aim was to design a study protocol for evaluating the efficacy of an interactive podcast program to assist Type 1 diabetes patients transitioning from adolescence to young adulthood.

**Design:** The study design is a parallel 1:1 two-arm randomized controlled trial emphasizing treatment fidelity through standardized interventions and improved adherence to reduce biases in outcomes.

**Methods:** This theoretical-based study will be conducted at two medical centers in northern Taiwan, enrolling 88 participants. Participants will be randomly assigned to either the experimental group, receiving the interactive podcast program “Living With Type 1 Diabetes to Grown-Up,” or the active control group, receiving the e-book “Transitioning from Adolescence to Early Adulthood: The Ins and Outs of Type 1 Diabetes.” The 3-month intervention will release 36 podcast episodes at a rate of three per week. Data will be collected at baseline, postintervention, and at 3 and 6 months postintervention to evaluate the efficacy of the intervention on disease control outcomes, emotional distress, diabetes knowledge, self-care behaviors, self-management confidence, interpersonal distress, and family conflict.

**Conclusion:** This first evidence-based, rigorously designed podcast program will offer valuable guidelines for future interventions aimed at helping adolescents with Type 1 diabetes transition to adulthood.

**Impact:** This study's positive findings could support podcasts as an innovative tool for helping adolescents with Type 1 diabetes transition to young adulthood. Additionally, it may provide valuable insights for future research and health policymakers, potentially transforming diabetes management approaches.

**Reporting Method:** The authors adhered to CONSORT guidelines to ensure transparency and reliability.

**Patient or Public Contribution:** There is no patient or public contribution.

**This Paper Contributes to the Wider Global Clinical Community:** This paper provides an evidence-based framework for using podcasts to support self-management and well-being in adolescents with Type 1 diabetes and offers insights for future digital health strategies.

**Trial Registration:** ClinicalTrials.gov identifier: NCT06464640

## 1. Introduction

Although Type 1 diabetes (T1D) accounts for only around 5%–10% of all diabetes cases, its global incidence and prevalence are increasing [1]. According to the US SEARCH for Diabetes in Youth study, T1D incidence was 22.2 per 100,000 in 2018, reflecting a 2.02% increase from 2002 [2]. In 2021, approximately 8.4 million people worldwide lived with T1D, including 1.5 million individuals under the age of 20 [3], with the highest incidence observed between the ages of 10 and 14 [4]. In Taiwan, T1D is most prevalent among individuals aged 0–19, with an incidence rate of 5.17 per 100,000 and a prevalence rate of 0.05% [5]. Given the significant global prevalence of T1D among children and adolescents, healthcare systems must be prepared to address the increasing needs of individuals transitioning from adolescence to early adulthood.

### 1.1. Background

Briefly, the transition from late adolescence to early adulthood is a critical developmental period marked by rapid physiological and psychological changes [6]. It is one of the most stressful periods in human development because it involves significant life experiences, such as making long-term decisions about relationships and careers, leading to uncertainty and fear [6, 7]. This is particularly true for individuals with chronic illnesses, as they must take on greater responsibility for self-care and disease management [8, 9]. Maintaining glycemic control during this period is especially challenging, with only 17% of patients achieving the glycemic control targets set by the American Diabetes Association [10]. Poor glycemic control can increase the risk of micro- and macrovascular complications [11], diabetic ketoacidosis hospitalizations [12], mortality [13], and heightened anxiety [14], adversely impacting sleep and quality of life [15, 16]. Therefore, recognizing the challenges in disease management for T1D patients during their transition period and developing interventions tailored to their needs is critical.

Technology is a key healthcare need of T1D patients aged 16–25 during their transition [17]. Participants emphasize its importance in managing their T1D [14, 17]. Technology-based interventions, such as blood glucose monitoring apps, text messaging, and online educational platforms, have been applied in managing T1D in young people [18, 19]. These interventions have shown significant or potential benefits, such as improved disease control outcomes [19, 20], reduced emotional distress [18], enhanced diabetes knowledge and self-care behaviors [19, 20], increased self-management confidence [18, 19], and decreased interpersonal distress [19] and family conflict [21]. However, inconsistent effects on blood glucose control (HbA1c) and psychological health outcomes [18–20] have also been reported. Lower study quality may have limited the ability of these studies to detect changes [19, 20]. Most importantly, these interventions were not tailored to individual needs and preferences [19].

mHealth applications have become popular among the younger tech-savvy generations, offering innovative approaches to managing chronic conditions and health behaviors [22–25] with the potential to be tailored to individual needs. In Taiwan, 98% of high school students use smartphones, with an average weekly usage of 6.39 days [24]. The proliferation of digital tools has spurred the growth of digital technology, with podcasts being one of the fastest growing digital educational media among adolescents [26]. In the United States, approximately 57% of individuals aged 12 and above have listened to podcasts, with an average weekly listening time of over 6 h [27]. In Taiwan, 16.3% of Internet users regularly listen to podcasts [28], presenting an opportunity to leverage podcast-based interventions to disseminate information [29] to T1D patients during transition.

### 1.2. Existing Literature and Gaps

Podcasts introduced in the early 2000s have gained widespread popularity. Creators upload programs to podcast platforms for on-demand streaming or downloading, offering a mobile platform that supports multitasking [30, 31]. They offer flexible and enjoyable learning experiences that can overcome temporal and spatial constraints, reduce anxiety, enhance motivation, and lead to effective learning outcomes [32–34]. Recently, podcasts have been increasingly integrated into healthcare education to improve clinical practice competencies [35, 36], enhance health-related nutritional literacy among high school students [37], improve parents' ability to assess the trustworthiness of treatment benefit claims [38], and address the complex educational needs of cancer survivors [39].

In the context of diabetes care, existing literature mainly addresses healthcare professionals, outlining treatment guidelines [40], exploring diabetes treatment trends [41], highlighting the importance of language in patient–provider communication for diabetes self-management [42], offering strategies to empower patients [43], and discussing recent advancements in diabetes research [44–46]. Recently, podcasts have started incorporating the perspectives of individuals with Type 2 diabetes [47] presenting as episode transcripts. However, a significant gap exists in using podcast-based interventions to support T1D patients during their transitional phase and improve their disease management and outcomes.

### 1.3. Theoretical Framework

The transition from adolescence to early adulthood involves significant life changes and environmental shifts. According to transition theory, these changes are recognized as antecedents and categorized as developmental, situational, health-illness, and organizational [48, 49]. Developmental transitions occur as individuals progress through different stages of human development. Situational transitions involve changes in context, such as education or occupation, resulting in shifts in personal roles or relationships. Health-illness transitions refer to changes in health status. Organizational transitions involve changes in institutional settings, such as care models. Nursing interventions are critical during these transitions, helping individuals achieve desired outcomes, including well-being (physical and psychological comfort), mastery (an individual's ability to manage and control their new situation or condition effectively), and relationship comfort (the quality and stability of family relationships) [49].

In this study, we focus on adolescents transitioning into early adulthood, following the framework of Chick and Meleis [48] and Schumacher and Meleis [49], identifying four types of transitions: developmental, situational, health-illness, and organizational. The intervention utilizes an interactive podcast program, “Living with Type 1 Diabetes to Grown-Up,” which explores outcomes of well-being (disease control outcomes and emotional distress), mastery (diabetes knowledge, self-care behaviors, and self-management confidence), and relationship comfort (interpersonal distress and family conflict).

**Figure 1:**
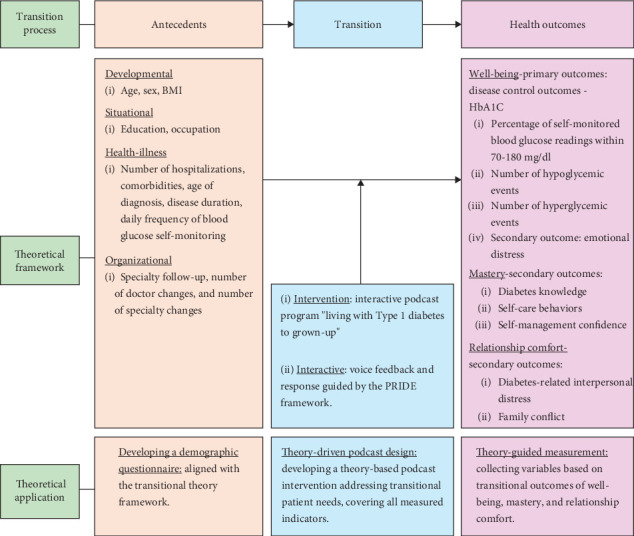
Theoretical framework of the study.

The interactive component of the intervention applies the self-regulation theory's PRIDE steps—problem identification, research into daily activities, goal identification, plan development, and self-reward mechanisms—commonly used in adolescent and disease self-management research [50]. Participants provide audio feedback according to study guidelines and steps, with downloadable responses from the research team.  Figure 1 illustrates the theoretical framework.

## 2. The Study

### 2.1. Aims

This study will implement rigorous treatment fidelity (TF) monitoring to evaluate the efficacy of the interactive podcast program, “Living with Type 1 Diabetes to Grown-Up” on disease control outcomes, emotional distress, diabetes knowledge, self-care behaviors, self-management confidence, interpersonal distress, and family conflict at baseline, postintervention, and 3 and 6 months postintervention.

### 2.2. Hypotheses

Based on the prior efficacy of apps and internet-based interventions [18, 19, 21], we hypothesized that participants in the experimental group receiving the interactive podcast program, “Living with Type 1 Diabetes to Grown-Up,” would demonstrate better disease control outcomes, along with improved diabetes knowledge, self-care behaviors, and self-management confidence, compared to those in the active control group. We also hypothesized that emotional distress, interpersonal distress, and family conflict would be lower in the experimental group compared to the active control group.

### 2.3. Developing the Podcast Intervention

From August 2023 to July 2024, the podcast intervention was developed and tested using the design science research framework of Hevner [51], emphasizing a user-centered approach. The development process comprised three key cycles: relevance, rigor, and design. The relevance cycle was shaped by insights from previous qualitative studies [14] and a Delphi study [17]. The rigor cycle involved an extensive literature review, and the results from both prior cycles were integrated into the design cycle. Content creation adhered to international quality guidelines for podcast production [52]. Usability was assessed through heuristic evaluation [53], think-aloud protocols [54], and measures of user interaction satisfaction [55], resulting in a final satisfaction score of 4.86 (± 0.33) [56]. The findings from these preliminary studies informed the development of the intervention, as detailed in Section 3.4.

## 3. Methods

### 3.1. Study Design

**Figure 2:**
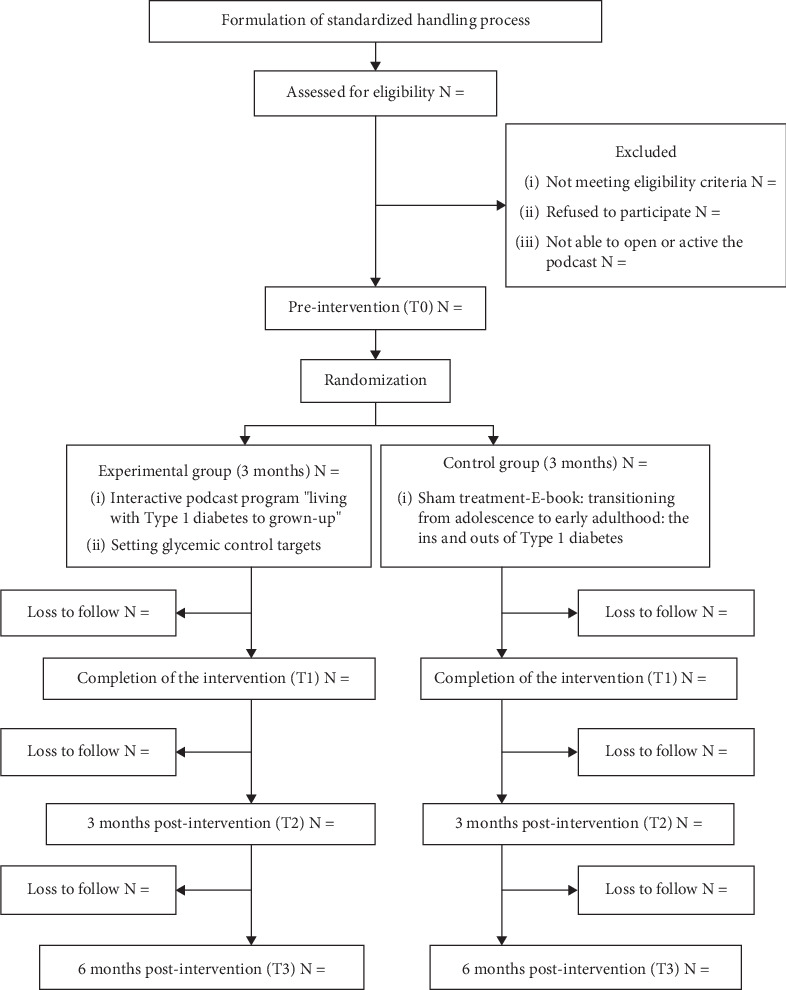
CONSORT diagram.

This study is a double-blind, parallel, 1:1 randomized controlled trial (RCT). After enrollment assessments, participants will be randomly assigned to either the experimental group, receiving the interactive podcast program, “Living with Type 1 Diabetes to Grown-Up,” or the active control group, receiving a sham treatment using an ineffective e-book, “Transitioning from Adolescence to Early Adulthood: The Ins and Outs of Type 1 Diabetes” which will be given at enrollment [57]. Both interventions will last for 3 months. Outcome measures will be evaluated at four time points: pre-randomization (T0), immediately postintervention (T1), 3 months post-intervention (T2), and 6 months postintervention (T3). This study has been registered at ClinicalTrials.gov (under identifier NCT06464640).  Figure 2 shows the detailed study process.

### 3.2. Study Setting

Participants will be recruited from the pediatric and adult endocrinology outpatient clinics and wards of two medical centers in northern Taiwan specializing in youth T1D treatment. The combined annual patient pool at these centers includes approximately 350 eligible candidates. We anticipate approaching approximately four eligible patients per week, with an expected refusal rate of 20% following the COVID-19 pandemic to recruit 12–16 participants per month [57]. We aim to recruit 88 participants (as per the sample size calculation detailed below) within a maximum of 6–8 months. Should unforeseen challenges, such as another public health crisis, arise, we will extend the recruitment period or explore additional recruitment sites as contingency measures.

### 3.3. Participants

#### 3.3.1. Eligibility Criteria

Participants will be recruited using convenience sampling. Inclusion criteria are as follows: (1) diagnosed with T1D by an endocrinologist before the age of 16, with a disease duration of more than 6 months, allowing sufficient time for diagnosis stabilization and self-care training [58, 59]; (2) aged between 16 and 25, representing the transition period from late adolescence to early adulthood [58, 60]; (3) mean HbA1c level ≥ 7.5% in the year before inclusion to identify patients who have inadequate blood sugar control, making them suitable candidates for the podcast intervention; (4) the ability to communicate in Mandarin or Taiwanese; (5) possession of a smartphone, tablet, or computer with Internet access, enabling program transmission and voice interaction for data collection; and (6) willingness to participate in the study with signed informed consent. For minors, the legal guardian must also provide signed informed consent. Individuals with other metabolic diseases, chromosomal abnormalities, major illnesses, or significant auditory and cognitive impairments will be excluded from this study, as these conditions may affect their healthcare needs and influence their understanding and utilization of the intervention [61, 62]. Participants using continuous glucose monitoring (CGM) will also be excluded from the trial, as its adoption remains low in Taiwan due to high out-of-pocket costs despite partial reimbursement [63]. This ensures consistency in glycemic data sources.

#### 3.3.2. Sample Size Calculation

Few studies have explored podcast-based interventions for chronic diseases or illnesses in adolescents. As podcasting is a technology-based intervention method, results from our prior study of Healthcare CEO App [57] were used for sample size calculation. Effect sizes were calculated using Cohen's *d*. For groups of 19 participants each, the mean HbA1c for the experimental vs control group at 3, 6, and 9 months postintervention was 7.93 ± 1.36 versus 8.69 ± 2.40, 8.30 ± 1.29 versus 8.82 ± 2.69, and 8.24 ± 1.58 versus 8.87 ± 2.72, respectively, yielding an effect size of 0.38, 0.26, and 0.32, respectively.

Using G-Power 3.1.9.4 software, the required sample sizes were calculated for each group across the three time points as 18, 38, and 26 participants, respectively. Assumptions included two groups with four repeated measures, *α* set at 0.05, power at 0.80, and a correlation coefficient of 0.5. No participants withdrew from our prior study [57]. However, considering a conservative dropout rate of 12% based on similar online intervention studies with a 6-month follow-up period [64], the number of participants was increased to 21, 44, and 30 per group. The maximum sample size calculated based on the smallest effect size at T2 was chosen. Therefore, the total sample size will be 88 participants.

### 3.4. Randomization and Blinding

A statistician not involved in data collection will use SPSS to randomly assign participants, with the results enclosed in sealed envelopes. After baseline assessment, envelopes will be opened, and group assignments (experimental or active control) will be disclosed to the interventionist via phone only.

Double blinding will be employed for data collectors and participants. Participants will be blinded through a sham treatment (see below), and data collectors will not have access to group assignments. To assess the effectiveness of the blinding, data collectors will be asked two questions at the start and conclusion of the study: “Are you aware of the interventions in this study?” and “Can you identify whether the participant was in the experimental or control group based on any clues?” Participants will also answer two questions: “What guidance did you receive during the study?” and “Do you think you were in the experimental or active control group?” These questions will help evaluate the success of the blinding process.

### 3.5. Intervention and Sham Treatments

#### 3.5.1. Experimental Group: Intervention

The intervention comprises the interactive podcast program, “Living with Type 1 Diabetes to Grown-Up,” which includes podcast episodes and interactive voice feedback. The program features 36 episodes, released at a rate of three per week over a 3-month intervention period [65]. Based on pilot testing for optimal duration and speech rate [56], each episode lasts 8–15 min with a word count of 160–220 words per minute. The content incorporates participant recommendations and uses appropriate language, presented lightly and engagingly. The topics and subtopics are detailed in Table 1. In addition to episode ratings and encouraged comments, participants will provide voice feedback aligned with the objectives and content of each unit to enhance practical application. During the first 9 weeks of the intervention, every 3 weeks (at Weeks 3, 6, and 9), participants will select a topic from the podcast episodes they have completed and provide voice feedback based on the PRIDE interactive voice feedback protocol and steps, as detailed in Table 2. The principal investigator will then coordinate expert responses tailored to participants' feedback, delivered in the following weeks 4, 7, and 10. Each participant will engage in three personalized voice feedback interactions over the study period, and all feedback will be thoroughly documented to assess TF through content checking, ensuring accurate implementation and alignment with participant needs. While episodes cannot be downloaded, participants can listen to them again throughout the intervention.

Detailed information on the training of interventionists is contained in File S1. Trained interventionists will follow the standard manual to (1) install the podcast program for the experimental group at enrollment by setting up participant codes and accounts; (2) explain the weekly release of three podcast episodes through dynamic, time-limited QR codes and guide participants through the process of submitting and retrieving voice feedback; (3) demonstrate the steps for inputting, uploading, and downloading voice feedback from the research team, followed by participant practice; (4) provide contact information for administrative support and address any related inquiries; (5) collaborate with participants to discuss and set HbA1c control goals at every data assessment point; and (6) facilitate and ensure treatment receipt and enactment throughout the intervention period (see Section 3.6 for detailed plans).

#### 3.5.2. Active Control Group: Sham Treatment

Our previous study demonstrated that e-books did not have a significant impact on glycemic control (HbA1c: 8.30 ± 1.29 vs. 8.82 ± 2.69, *p* = 0.477), diabetes knowledge (16.59 ± 3.26 vs. 15.42 ± 3.85, *p* = 0.336), self-care behaviors (152.94 ± 13.38 vs. 155.95 ± 14.56, *p* = 0.525), self-management confidence (29.82 ± 4.30 vs. 27.63 ± 5.82, *p* = 0.212), or interpersonal distress (11.00 ± 5.01 vs. 10.53 ± 6.54, *p* = 0.811) after 6 months in our prior trial [57]. Therefore, the active control group will receive the e-book “Transitioning from Adolescence to Early Adulthood: The Ins and Outs of Type 1 Diabetes,” compiled from the Healthcare CEO app collection, as a sham treatment. This title and content indicate its suitability as a minimally active control that is thematically relevant [66, 67]. The topics in the e-book include (1) diabetes knowledge, (2) how to manage your blood sugar, (3) eating and exercising well, (4) healthy interpersonal relationships, (5) parent–child harmony, and (6) you are not alone. Each unit includes recommended readings, such as “Needles” and “My Life Is Not Controlled by Diabetes.” Although parts of the themes are similar, the e-book is simplified to 600–800 words of pure text per unit, lacking T1D patient experience sharing and interactive engagement. Participants in the active control group will not be reminded to read the e-book, no additional interventions or consultations will be provided, and no follow-up calls will be made during the intervention. After the study, participants in the active control group will be offered the opportunity to listen to all podcast episodes and receive the same incentives as the experimental group, if desired. The intervention characteristics of the experimental and active control groups are presented in Table 3.

### 3.6. TF

To ensure the fidelity and accuracy of the intervention process and enhance its effectiveness, this study adheres to the five components outlined by the National Institute of Health Behavior Change Consortium: treatment design, provider training, treatment delivery, treatment receipt, and treatment skills enactment [68]. Furthermore, insights from Saarijärvi et al. [69] on training research assistants and backend data analysis are also incorporated.  Table 4 presents a detailed TF strategy, while File S1 provides the relevant manuals and checklists.

#### 3.6.1. Strategies for Enhancing Treatment Delivery and Receipt

To ensure appropriate treatment delivery and receipt, the trained interventionists will send reminders on Wednesdays if backend data shows that participants have not listened to the episodes. Additionally, 1.5 months postintervention, the assistant will contact participants by phone to check for any issues or difficulties with using the podcast without discussing diabetes care–related information to avoid study interference [57]. After the intervention, the administrator will disable the program link from the backend.

#### 3.6.2. Strategies for Enhancing Participant Involvement

Based on the findings from an earlier study [56], strategies to boost participant engagement will be adopted at the treatment design, delivery, receipt, and enactment stages to include (1) increasing the willingness to engage with the podcast program: (A) tailoring the content to resonate with youth culture and language, based on participants' input. (B) Employing professional visual design for the podcast program cover to captivate participants both visually and audibly. An art designer with expertise in nursing and experience with T1D was enlisted to create an appealing cover for the target age group. (C) Enhancing participant engagement through personalized feedback and voice interaction. (D) Encouraging participants to leave voice messages and ratings after each episode. Following the study, the research team will review the messages and select five participants with the most relevant contributions, awarding them a voucher worth NT$300 each. (2) Offering NT$300 vouchers to participants for each completed questionnaire as a token of appreciation. (3) Establishing HbA1c achievement goals, with the Top 5 participants showing the highest percentage improvement receiving NT$500 vouchers at the study's conclusion as an incentive.

### 3.7. Outcomes

The study utilizes structured questionnaires, accessible via QR code, for data collection. All questionnaires are mandatory and have been validated by experts. The estimated completion time is 15–20 min. A detailed description of the psychometric properties of each questionnaire used in this study is available in File S1.

#### 3.7.1. Basic Information Questionnaire

This questionnaire covers age, gender, body mass index, education, occupation, number of hospitalizations, comorbidities, age of diagnosis, disease duration, daily frequency of blood glucose self-monitoring, specialty follow-up, and number of doctor and specialty changes.

#### 3.7.2. Primary Outcome: Wellbeing—Disease Control Outcomes

Assessed through HbA1c, the percentage of self-monitored blood glucose readings within the 70–180 mg/dL range, the number of hypoglycemic events (blood glucose < 70 mg/dL), and the number of hyperglycemic events (blood glucose > 180 mg/dL) [70] will be noted. HbA1c will be recorded from electronic medical records after blood samples are collected during patient visits. The percentage of self-monitored blood glucose readings within the 70–180 mg/dL range will be calculated using the formula: (number of values within the 70–180 mg/dL range/total number of tests)∗100% [57].

A data downloader at outpatient clinics will be used to retrieve the past 3 months' blood glucose data and reset it to prevent data from overlapping or being overwritten. Subsequently, two data collection assistants will simultaneously review the data manually. If discrepancies arise, the principal investigator will verify the data. Finally, interrater reliability will be assessed using Cohen's Kappa coefficient to ensure consistency in their evaluations [71]. In cases of abnormal blood glucose values, such as hypoglycemia, if the participant follows up with a blood glucose check 15 min after consuming carbohydrates, only the first recorded value (i.e., the pretreatment blood glucose value) will be used for analysis. Commercial blood glucose meters can store 500–1000 data points, and participants typically measure 2–5 times daily. Therefore, based on prior studies, data coverage is not expected to be an issue [57].

#### 3.7.3. Secondary Outcome: Well-Being—Emotional Distress

This outcome will be measured using the Chinese version of the Diabetes Distress Scale. Three subscales with 15 items will be used: emotional burden, physician-related distress, and regimen-related distress; the fourth subscale, interpersonal distress, will measure “Relationship Comfort: Diabetes-Related Interpersonal Distress” in this study. Total scores range from 15 to 60, with higher scores indicating more significant distress [72].

#### 3.7.4. Secondary Outcome: Mastery—Diabetes Knowledge, Self-Care Behaviors, and Self-Management Confidence

Diabetes knowledge will be measured using the Chinese version of the Diabetes Knowledge Questionnaire comprising 24 items. Participants must answer “Yes,” “No,” or “I don't know” for each item and are awarded 1 point for each correct answer. Total scores range from 0 to 24 points, with higher scores indicating more knowledge of diabetes [73]. The original questionnaire was developed for Type 2 diabetes, but Items 6 and 13 have been modified for relevance to T1D. Item 6, “If I have diabetes, my children are very likely to have diabetes,” is changed to “false,” and Item 13, “Medication therapy controls diabetes better than appropriate diet and exercise,” is changed to “true.” These modifications have been validated in T1D patients [57].

Self-care behaviors will be measured using the Diabetes Self-Care Behavior Scale comprising 39 items under seven dimensions: medication management (insulin injection), healthy eating (diet control), blood glucose monitoring (self-monitoring), physical activity (regular exercise), problem-solving, risk reduction, and healthy coping (psychosocial adaptation and stress management). Total scores range from 39 to 195, with higher scores indicating superior self-care [74].

Self-management confidence will be measured using the Chinese version of the Perceived Diabetes Self-Management Scale comprising 24 items. Total scores range from 8 to 40, with higher scores indicating higher confidence in diabetes self-management [75].

#### 3.7.5. Secondary Outcome: Relationship Comfort—Diabetes-Related Interpersonal Distress and Family Conflict

Diabetes-related interpersonal distress will be measured using the Chinese version of Interpersonal Distress, a subscale of the Diabetes Distress Scale with three items: “Feeling that friends or family are not supportive enough of my self-care efforts (e.g., planning activities that conflict with my schedule, encouraging me to eat the “wrong” foods),” “Feeling that friends or family do not appreciate how difficult living with diabetes can be,” and “Feeling that friends or family do not give me the emotional support that I would like.” Total scores range from 1 to 12, with higher scores indicating more significant diabetes-related interpersonal distress [72].

Family conflict will be measured using the Diabetes Family Conflict Scale-Revised comprising 19 items under two dimensions: direct and indirect management tasks. Direct management tasks refer to conflicts directly related to diabetes care, such as “During the past month, I have argued with my parent(s) about the results of blood sugar monitoring.” Indirect management tasks refer to conflicts not directly related to diabetes care, such as: “ During the past month, I have argued with my parent(s) about telling friends about diabetes.” Total scores range from 19 to 57, with higher scores indicating more significant conflict [76].

### 3.8. Statistical Analysis

Descriptive statistics such as frequency distribution, percentage, mean, standard deviation, and maximum/minimum scores will be utilized to characterize the subjects' demographics, disease characteristics, outcome variables at enrollment, and podcast usage. The efficacy of the intervention will be assessed using generalized estimating equation (GEE) modeling, which accommodates repeated measurements and deals with missing data effectively, following the intention-to-treat principle.

GEE will evaluate differences in outcome variables between experimental and control groups over time. Linear GEE models will be applied for all the continuous outcomes. The models will include fixed effects for the group (experimental vs. control), time (baseline, postintervention, and 3 and 6 months postintervention), and their interaction. The working correlation structure will be selected based on the quasi-likelihood under the independence model criterion [77]. Statistical significance will be determined at *p* < 0.05. All analyses will be conducted using IBM SPSS 26.0 for Windows.

### 3.9. Podcast Voice Feedback Data Analysis

Thematic categorization and calculation of theme occurrence frequencies will be conducted to support process evaluation, particularly in understanding participant engagement and informing intervention fidelity.

### 3.10. Ethical Considerations

The study was approved by the Chang Gung Medical Foundation Institutional Review Board (Submission Reference: 202300552B0C601) on April 14, 2024, under the Declaration of Helsinki guidelines.

### 3.11. Validity and Reliability/Rigor

To maximize the validity and reliability of the study, we enhanced the intervention fidelity following the NIH Behavior Change Consortium [68] (see Section 3.6). The trial will follow the CONSORT 2010 guidelines [78].

## 4. Discussion

### 4.1. Strengths

This study will evaluate the efficacy of the interactive podcast program “Living with Type 1 Diabetes to Grown-Up” using a double-blind, parallel 1:1 RCT. The program has several strengths. First, its development is theory-based [51] and tailored to the healthcare needs of T1D patients in transition [14, 17], employing a user-centered approach for evaluation and testing [53–55]. The content, number of episodes, frequency, duration, and language are all designed to meet patients' expectations during this transition [56]. Second, the program's immediate personalized interaction through voice feedback enhances information exchange by avoiding traditional text or photo communication. This voice messaging approach improves content review, applicability to daily life, and professional support, increasing patients' motivation and willingness to engage [79]. This feature sets the program apart from existing podcasts, audiobooks, or certain YouTube content. Finally, TF is a significant factor affecting the efficacy of RCTs [80]. Therefore, both the experimental and control groups will implement methods to standardize treatments received and optimize TF, enhancing the credibility of the study findings [80]. Furthermore, in addition to the rigorous RCT design, this study has developed strategies to enhance TF based on existing literature and increased the number of research assistants. Comprehensive classroom and clinical shadowing training will ensure effective TF monitoring (File S1). These strategies allow for a more comprehensive analysis of the intervention's efficacy and help reduce bias in the study results.

### 4.2. Limitations

This study also has several limitations. The intervention delivery and voice feedback rely on Internet access, which may be influenced by factors such as accessibility, bandwidth, and data traffic capacity. These issues could affect participants' experience and level of engagement, a common challenge in technology-based interventions [81]. However, a feasibility test of the interactive podcast program indicated that participants were able to log in and complete the listening sessions on time [56]. The intervention frequency is set at thrice a week, with each session lasting 8–15 min. This schedule provides flexibility for participants to engage with the program and offer feedback, helping to mitigate the impact of Internet limitations to some extent. Additionally, maintaining participant engagement can be challenging. To address this, the study will analyze participants' login and listening behavior after program release, provide reminders as needed, and offer personalized voice feedback and appropriate reward strategies each month to enhance patient engagement. Moreover, the use of CGM is increasing globally, offering benefits like better glycemic control and psychosocial well-being [82, 83]. However, in Taiwan, adoption is limited due to high out-of-pocket costs, despite partial reimbursement, with about 1692 procedures performed annually, regardless of age [63]. As a result, this study excluded CGM users to ensure consistent outcome assessments, which may limit the generalizability of the findings to populations with better access to CGM technology. In regions where CGM use is prevalent, future program adaptations may integrate CGM-derived metrics like time-in-range as main outcomes. Additionally, CGM data could enable personalized voice feedback based on real-time glycemic patterns, helping participants identify self-management challenges, track progress, and enhance the PRIDE feedback loop for better engagement and targeted interventions. Finally, the podcast program is specifically tailored for T1D patients in transition in Taiwan. While the PRIDE self-regulation steps and the youth-centered structure may be applicable across various contexts, certain elements necessitate adaptation. For instance, family involvement, dietary practices, and communication styles with healthcare providers differ by region and should be tailored accordingly [84, 85].

## 5. Conclusion

This first interactive podcast program, “Living with Type 1 Diabetes to Grown-Up” developed based on theories for T1D patients in transition will start to recruit participants from November 2024. With rigorous RCT design and TF monitoring strategies in place, expected efficacy on disease control, emotional distress, diabetes knowledge, self-care behaviors, self-management confidence, interpersonal distress, and family conflict will provide valuable evidence supporting the use of podcasts to assist patients in transition.

## Figures and Tables

**Table 1:** Podcast program schedule.

**Units**	**Mechanism (targets)**	**Weeks**	**Episode title (contents/corresponding to PRIDE steps)**
Opening	Introducing the topic of T1D aimed at: 1. Providing general disease knowledge 2. Enhancing disease knowledge and proper understanding of T1D	1	EP1: The birth of the interactive podcast program “Living With Type 1 Diabetes To Grown-Up”
			EP2: The story of diabetes—origins and current status (distinguishing different types of diabetes and fostering a proper understanding of T1D/P, R, I)
			EP3: The story of diabetes—evolution of treatment (distinguishing different types of diabetes and fostering a proper understanding of T1D/P, R, I)

Approaching adulthood (transitioning into adulthood)	Discussing issues related to changes and management of T1D during late adolescence to early adulthood aimed at: 1. Enhancing transitional T1D knowledge and management strategies. 2. Building confidence in T1D management through accumulated knowledge, improving self-care behaviors, glycemic control outcomes, and reducing diabetes-related emotional distress.	2	EP4: Out of-control T1D (causes of unstable blood glucose during adolescence and related complications/P, R)
			EP5: I will conquer you (content and goals of blood glucose self-management during transition/I)
			EP6: Tips for living with T1D (incorporating T1D care into daily life/D, E)
		3	EP7: exercise is essential (relationship between exercise and blood glucose/P, R, I)
			EP8: Are you eating right? (appropriate dietary management/P, R, I)
			EP9: Exercise and consume, no concern! (strategies for exercise and dietary management/D, E)
		4	EP10: Stress, blood glucose, and sleep (impact of stress on blood glucose and sleep during transition/P, R, I)
			EP11: T1D and COVID-19 (impact of COVID-19 on T1D/P, R I)
			EP12: Making peace with stress (stress management/D, E)
		5	EP13: Loopy…((impact of substance use like tobacco, alcohol, drugs onT1D management/P, R, I))
			EP14: Sexuality and reproduction (T1D and issues related to sexuality and heredity/P, R, I)
			EP15: Behavior without blunder (strategies to avoid risky behaviors and unnecessary worries/D, E)
		6	EP16: Uncertain future (concerns about T1D, education, and employment/P, R, I)
			EP17: Transition to adult care? (issues related to transitioning care models/P, R, I)
			EP18: My future is not a dream (planning for future education, employment, and healthcare/D, E)

Sense it and say it right	Discussing potential communication issues during transition, aimed at: 1. Improving communication skills, enhancing the ability to cope with interpersonal distress, reducing interpersonal distress and family conflict	7	EP19: To tell or not to tell?—friends edition (concerns and issues in communicating with friends and colleagues/P, R, I)
			EP20: To tell or not to tell?—relationship edition (concerns and issues in communicating with partners/P, R, I)
			EP21: How to express (communication skills for relationships, friends, and workplace/D, E)
		8	EP22: Love amidst conflict (common causes of family conflict/P, R, I)
			EP23: Whose responsibility is it? (transfer and assignment of care responsibilities/P, R, I)
			EP24: Let us do it together (achieving consensus and gradually sharing/assuming care tasks/D, E)

Racing towards the sunshine	Discussing potential emotional distress during transition, aimed at: 1. Learning coping strategies to reduce emotional distress caused by emotional burden	9	EP25: quieting the limbic system (emotional distress caused by T1D/P, R, I)
			EP26: Sleeping well (T1D and sleep issues/P, R, I)
			EP27: Say yes to yourself! (coping with emotional distress and sleep issues, living authentically/D, E)

Diabetes talk from the dia-buddies	Inviting T1D peers to share their experiences, aimed at: 1. Providing role models to enhance disease management skills, increase self-care motivation, and improve disease control outcomes	10	EP28: Inviting a peer (sharing experience of being defeated by diabetes/P, R, I, D, E)
			EP29: Inviting a peer (sharing experience as a parent/PRIDE)
			EP30: Inviting a peer (sharing experience of coping with emotional distress/P, R, I, D, E)
		11	EP31: Inviting a peer (sharing experience of interpersonal communication/P, R, I, D, E)
			EP32: Inviting a peer (sharing experience of responsibility transition/P, R, I, D, E)
			EP33: Inviting Dr. Lin Chia-Hung (discussing effective blood glucose control and managing T1D as a manageable condition/P, R, I, D, E)

Looking forward to our next aerial encounter	Reviewing critical points aimed at: 1. Helping patients feel they are not alone and reflecting on their journey through others' experiences 2. Summarizing and highlighting the critical points of the program	12	EP34: We went through it together—Part 1 (summary and sharing of PRIDE interaction records)
			EP35: We went through it together—Part 2 (summary and sharing of PRIDE interaction records)
			EP36: Key point review and temporary goodbye (reviewing critical points of the related topics)

**Table 2:** PRIDE voice feedback guidelines.

**Steps of voice feedback**	**1. P: Problem—identify the primary problem**	**2. R: Research—review daily activities**	**3. I: Identify—set specific, achievable health management goals**	**4.D: Develop—develop a goal-achievement plan**	**5. E: Establish—develop a self-reward approach**	**6. Self-reflection on goal achievement status**
The first	After listening to Weeks 1–3, identify one of your most significant difficulties in disease understanding, blood glucose management, exercise, and diet (one primary issue). How does this problem impact your health management? For example, frequent post-exercise hypoglycemia, fear of not waking up, and avoiding exercise lead to poorer blood glucose control.	Reflect on the past 3 weeks and applying program knowledge, such as exercise, is essential to investigate your daily activities and identify the causes of the previous problem. For example, there is no habit of checking blood glucose before and after exercise or not knowing how to replenish carbohydrates during exercise.	Based on Weeks 1–3 of learning, set specific health management goals to address the issue. For example, no postexercise hypoglycemia after regular aerobic exercise for three consecutive weeks exists.	List and apply strategies learned from the program to achieve your goals. For example, monitor exercise type and blood glucose changes before and after, analyze the timing of hypoglycemia, and carry juice for incremental carbohydrate intake.	Establish a reward for achieving your goal. For example, if I avoid post-exercise hypoglycemia for 3 weeks, I will treat myself to a healthy meal.	Did you achieve your goal? Reasons for success or failure?

Self-verification (check and upload)						
The second	After listening to Weeks 4–6, what are the main issues in stress management, behavior control, genetic knowledge, or future planning? How do these issues affect your health management?	Reflect on the past 3 weeks and apply program knowledge, such as stress, substance use, diabetes and sexuality, and uncertain future, to review your daily activities and identify the root causes of the main issues.	Based on Weeks 4–6 of learning, set specific health management goals to address the issue.	List and apply strategies learned from the program, such as stress management, to develop a plan for achieving your goals.	Establish a new reward system for achieving your goal. For example, if I practice bedtime meditation and go to bed on time for three consecutive weeks, I will reward myself with a concert.	Did you achieve your goal? Reasons for success or failure?

Self-verification (check and upload)						
The third	After listening to Weeks 7–9, what are the main issues in disease disclosure, interpersonal relationships, communication, and emotional distress? How do these issues affect your disease management?	Reflect on the past 3 weeks and apply program knowledge, such as concerns about interpersonal communication, common causes of family conflict, and resulting emotional distress, to identify the root causes of the main issues.	Based on Weeks 4–6 of learning, set specific health management goals to address the issue.	List and apply strategies learned from the program, such as stress management, to develop a plan for achieving your goals.	Establish a new reward system for achieving your goal. For example, if I achieve my planning and communication goals for these 3 weeks, I will reward myself with a relaxing trip.	Did you achieve your goal? Reasons for success or failure?

Self-verification (check and upload)						

**Table 3:** The intervention characteristics between the experimental group and active control group.

**Key differences in intervention**		**Experimental group (intervention)**	**Active control group (sham treatment)**
Intervention modality	Voice media	V	—
	Text-based E-book	—	V

Interaction	Voice feedback and response	V	—
	No interaction	—	V

Intervention topics	Knowledge	V	V
	Exercise and diet	V	V
	Interpersonal relationships	V	V
	Parent-child relationships	V	V
	T1D and COVID-19	V	—
	Sleep	V	—
	Stress	V	—
	Risk behaviors	V	—
	Sexuality and genetics	V	—
	Employment and career	V	—
	Transition to adult healthcare	V	—

Intervention mechanism	Behavior modification (PRIDE framework)	V	—
	Peer experience share	V	—
	Personalized professional guidance	V	—

Intervention contact	Phone contact at 1.5 months of intervention	V	—

**Table 4:** The strategies of treatment fidelity.

**Dimensions**	**Strategies**	**Contents**	**Target population**		
			**Research assistants**	**Experimental group (intervention)**	**Active control group (sham treatment)**
Treatment design	1.「Standard manual for experimental and active control groups」	(1) Study objectives and standardized data collection process	RA_1_	—	—
		(2) Study objectives and standardized intervention details, intervention implementation process, and interaction	RA_3_	—	—
	2. 「Experimental group and active control group checklist for intervention steps」	Audit and ensure the completeness and fidelity of interventions conducted by RA_1_ and RA_3_.	RA_2_&RA_4_	—	—

Training provider	1.「Research assistant training manual」	(1) Training by the PI for 40 h (2) Challenges and management of T1D patients in transition (3) Study protocol, execution methods, intervention or data collection procedures, considerations, and ethical issues (4) Communication and interaction skills	RA_1-4_	—	—
	2. Clinical shadowing training	(1) Observation: two clinic shadowing sessions for familiarization and demonstrations by experienced graduate students (2) Practice: the first 10 participants are handled with PI's support and discussion	RA_1-4_	—	—

Delivery of treatment	1. Concurrent data collection and intervention implementation by two research assistants	(1) Conduct data collection (RA_1_) or intervention implementation (RA_3_) according to the “standard manual” (2) Intervention audit according to the “checklist for intervention steps “by RA_2_ and RA_4_	RA_1-4_	—	—
	2. Intervention process analysis	Audio recordings of all sessions and analysis randomly by the PI	RA_1-4_	—	—
	3. Regular discussions	Logs for recording issues during data collection and intervention implementation; weekly discussions with the PI [69]	RA_1-4_	—	—

Receipt of treatment	1. Field discussion	Discussions and clarifications with participants during interventions	—	V	V
	2. Teach back	Participants demonstrate the intervention and receive feedback	—	V	V
	3. Intervention content checklist for participant	Participants confirm receipt of all intervention components after completion	—	V	V

Enactment of treatment skill	Random follow-up	Monthly random follow-up with 5 participants, analyzing login and feedback records to ensure implementation in daily life [69]	—	V	—

Abbreviations: RA_1_, Research Assistant 1, responsible for data collection; RA_2_, Research Assistant 2, supervising the data collection process; RA_3_, Research Assistant 3, responsible for intervention implementation; RA_4_, Research Assistant 4, auditing the implementation of interventions.

## Data Availability

This article describes a protocol, and data collection has not yet occurred. Once the study is complete, data will be available from the corresponding author upon reasonable request.

## Supporting Information

Supporting Information 1 Additional supporting information can be found online in the Supporting Information section. File S1: Supplement of TF and Questionnaire.docx. This file provides the manuals and tabular checklists for treatment fidelity (including the Standard manual for experimental and active control groups, the groups checklist for intervention steps, the research assistant training manual, and the intervention content checklist for participants), as well as detailed descriptions of the study scales. These tables complement the methods and enhance the transparency of the reported outcomes.
