# Supplementary material for: Efficacy of the Online Interactive Podcast Program “Living With Type 1 Diabetes to Grown-Up”: A Two-Arm Randomized Controlled Trial Protocol
Source: J Diabetes Res. 2025 Sep 13;2025:4866975. doi: 10.1155/jdr/4866975 (PMC12450109; doi:10.1155/jdr/4866975)
Supplement: Supporting Information 1 — Additional supporting information can be found online in the Supporting Information section. File S1: Supplement of TF and Questionnaire.docx. This file provides the manuals and tabular checklists for treatment fidelity (including the Standard manual for experimental and active control groups, the groups checklist for intervention steps, the research assistant training manual, and the intervention content checklist for participants), as well as detailed descriptions of the study scales. These tables complement the methods and enhance the transparency of the reported outcomes. [file 4866975.f1.docx]

**Supplementary File 1- Supplement of TF and Questionnaire**

**Supplement 1** Manual and checklists for treatment fidelity

| **Supplement 1-1: The Standard Manual for Experimental and Active Control Group** |
| --- |
|  |
| 1. **Experimental Group** |
|  |
| **Description:** |
|  |
| Participants will engage in the interactive podcast program titled "Transitioning to Adulthood with Type 1 Diabetes." The program includes 36 episodes, released over three months, with three episodes released weekly. In addition, participants will participate in three voice feedback sessions conducted within nine weeks. |
|  |
| **Components:** |
|  |
| **1. Podcast Episodes:** Each episode lasts 8-15 minutes, totaling 36 episodes (see Appendix 1). |
| **2. Voice Feedback Sessions:** Three voice feedback sessions are scheduled, with one session every three weeks. |
| **3. Monitoring and Reminders:** Weekly monitoring will check if participants have listened to the episodes. If episodes are not listened to, reminders will be sent. |
| **4. Procedures:** |
| **4-1 Assign Participant Codes and Accounts:** |
| Create unique codes and accounts for each participant to access the Podcast platform. |
| **4-2 Provide Download and Usage Instructions:** |
| Provide clear instructions on how to use the QR code to download and listen to the Podcast. Demonstrate the download process and how to replay episodes if needed. |
| **4-3 Explain Study Purpose and Process Again:** |
| Reiterate the study purpose and procedures to ensure participants fully understand the significance and requirements of their participation. |
| **4-4 Reconfirm Consent Form Completeness:** |
| Recheck that all participants have submitted the complete informed consent forms. |
| **4-5 Guide on Voice Feedback:** |
| Guide participants on how to provide voice feedback based on PRIDE steps (Problem identification, Routine evaluation, Goal setting, Development of a plan, and Execution and reward establishment). Explain how to upload their feedback to the platform and how to receive feedback from the research team. |
| **4-6 Monitor Listening and Feedback Submission:** |
| Weekly monitoring to check whether participants have listened to the Podcast and submitted feedback. |
| **4-7 Support and Clarify Technical Issues:** |
| Provide support for any technical issues participants may encounter when accessing or listening to the Podcast. Ensure all communication is limited to technical support only. |
| **4-8 Ensure Consistency in Communication:** |
| Maintain consistent terminology and language in all communications with participants to ensure standardization across the study. |
| **4-9 Data Collection Time Points:** |
| Data will be collected at the following time points: Baseline (T0), immediately post-intervention (T1), three months post-intervention (T2), and six months post-intervention (T3). |
|  |
| **Monitoring:** |
|  |
| **1. Weekly Monitoring:** Weekly checks of participants' Podcast listening status. |
| **2. Feedback Monitoring:** Every three weeks, check the status of voice feedback submission and provide feedback accordingly. |
| **3. Regular Contact:** Every 1.5 months, contact participants via phone to address potential technical or usage issues without discussing disease management. |
|  |
| **Compensation:** |
|  |
| Participants who complete the feedback sessions and achieve HbA1C targets will receive rewards. Examples include personalized feedback from professionals or gift vouchers. |
|  |
| 1. **Active Control Group** |
|  |
| **Description:** |
|  |
| Participants will read an E-book "Transitioning from Adolescence to Early Adulthood: The Ins and Outs of Type 1 Diabetes. over a period of three months. No additional interaction or reminders will be provided during this period. |
| **Components:** |
|  |
| **1. e-Book:** The e-book contains various topics including: |
| Knowledge about diabetes |
| How to manage blood sugar effectively |
| Healthy eating and exercise habits |
| Building and maintaining interpersonal relationships |
| Stress-free parent-child relationships |
| Emotional support and not feeling alone |
| The e-book also includes recommended additional readings, such as "Life Outside the Donut" and "My Life Is Not Controlled by Diabetes," accessible via QR code for three months. |
|  |
| **2. Procedures:** |
|  |
| **2-1 Assign Participant Codes and Accounts:** |
| Create unique codes and accounts for each participant to access the e-book. |
| **2-2 Provide Download and Usage Instructions:** |
| Provide clear instructions on how to use the QR code to download and access the e-book. Explain the process for navigating the e-book content and accessing the material throughout the three-month period. |
| **2-3 Explain Study Purpose and Process Again:** |
| Reiterate the study purpose and procedures to ensure participants fully understand the significance and requirements of their participation. |
| **2-4 Reconfirm Consent Form Completeness:** |
| Recheck that all participants have submitted the complete informed consent forms. |
| **2-5 Support and Clarify Technical Issues:** |
| Provide support for any technical issues participants may encounter when accessing or reading the e-book during the accessing process. Ensure all communication is limited to technical support only. |
| **2-6 Ensure Consistency in Communication:** |
| Maintain consistent terminology and language in all communications with participants to ensure standardization across the study. |
| **2-7 Data Collection Time Points:** |
| Data will be collected at the following time points: Baseline (T0), immediately post-intervention (T1), three months post-intervention (T2), and six months post-intervention (T3). |
|  |
| **No Additional Interventions or Interactions:** |
|  |
| No voice feedback, reminders, or other interactions will be provided during the three-month period, except for initial technical support related to accessing the e-book. |

| **Supplement 1-2** **The Groups Checklist for Intervention Steps**  **Experimental Checklist for Intervention Steps** | | | | | | | |
| --- | --- | --- | --- | --- | --- | --- | --- |
| **Code:** |  |  |  |  |  |  |  |
| **Step** | **Description** | **Score** | | | | |  |
|  |  | **0** | **1** | **2** | **3** | **4** | **Scoring Explanation** |
| 1. Reconfirm Eligibility Criteria | Did RA re-evaluate participants based on inclusion criteria to ensure all participants meet the study requirements? | 0 | 1 | 2 | 3 | 4 | 0 = Not confirmed; 1 = Partially confirmed; 2 = Mostly confirmed; 3 = Fully confirmed; 4 = Fully confirmed with no errors. |
| 2. Reconfirm Exclusion Criteria | Did RA re-evaluate participants based on exclusion criteria to ensure no participants meet the exclusion criteria? | 0 | 1 | 2 | 3 | 4 | 0 = Not confirmed; 1 = Partially confirmed; 2 = Mostly confirmed; 3 = Fully confirmed; 4 = Fully confirmed with no errors. |
| 3. Re-explain Study Purpose | Did RA re-explain the study purpose and procedures to participants to ensure they fully understand the significance of their participation? | 0 | 1 | 2 | 3 | 4 | 0 = No explanation; 1 = Incomplete explanation; 2 = Partially explained; 3 = Fully explained; 4 = Fully and clearly explained. |
| 4. Reconfirm Consent Form Completeness | Did RA recheck the completeness of the written informed consent forms for all participants? | 0 | 1 | 2 | 3 | 4 | 0 = Not confirmed; 1 = Partially confirmed; 2 = Mostly confirmed; 3 = Fully confirmed; 4 = Fully confirmed with no errors. |
| 5. Provide Instructions for Podcast Access | Did RA clearly explain how to use the QR code to download and listen to the Podcast, including release schedule and frequency? | 0 | 1 | 2 | 3 | 4 | 0 = Not provided; 1 = Incomplete guidance; 2 = Partially detailed guidance; 3 = Complete guidance; 4 = Detailed and specific guidance. |
| 6. Guide Initial Podcast Listening | Did RA guide participants to complete at least one Podcast download and listening session? | 0 | 1 | 2 | 3 | 4 | 0 = No guidance; 1 = Basic guidance; 2 = Partial guidance; 3 = Completed guidance; 4 = Completed guidance with no technical issues. |
| 7. Guide on Providing Voice Feedback | Did RA clearly inform participants about the schedule and frequency of voice feedback and guide them on how to provide feedback based on PRIDE guidelines? | 0 | 1 | 2 | 3 | 4 | 0 = No guidance; 1 = Partial guidance; 2 = Full guidance but unclear; 3 = Complete and clear guidance; 4 = Clear guidance addressing all questions. |
| 8. Explain and Demonstrate How to Obtain Professional Feedback | Did RA explain how participants can receive professional feedback, including methods and timing? | 0 | 1 | 2 | 3 | 4 | 0 = No explanation; 1 = Incomplete explanation; 2 = Partial explanation; 3 = Full explanation; 4 = Full and clear explanation of all methods and steps. |
| 9. Provide Contact Information for Staff | Did RA clearly explain how to contact the management team and suggest appropriate contact times? | 0 | 1 | 2 | 3 | 4 | 0 = No explanation; 1 = Incomplete explanation; 2 = Partial explanation; 3 = Complete explanation; 4 = Full and clear explanation of contact methods and timings. |
| 10. Explain Reminder Situations and Timing | Did RA explain the weekly check of participants' Podcast listening and the timing of reminders? | 0 | 1 | 2 | 3 | 4 | 0 = No explain; 1 = Partial explain; 2 = Mostly explain; 3 = Fully explain; 4 = Fully explain. |
| 11. Explain Regular Technical Support Contacts | Did RA inform participants about the scheduled contacts every 1.5 months to check for technical issues? | 0 | 1 | 2 | 3 | 4 | 0 = No inform; 1 = Partial inform; 2 = Mostly inform; 3 = Fully inform; 4 = Fully inform. |
| 12. Respond to Participants' Questions | Did RA appropriately address or clarify participants' questions during the Podcast intervention process? | 0 | 1 | 2 | 3 | 4 | 0 = No response; 1 = Partial response; 2 = Mostly responded; 3 = Fully responded; 4 = Full and clear response. |
| 13. HbA1C Goal Setting | Did RA discuss with the patient and set an appropriate HbA1C target? | 0 | 1 | 2 | 3 | 4 | 0 = No discuss; 1 = Incomplete discuss; 2 = Partial discuss; 3 = Full discuss; 4 = Full and clear discuss and set goal. |
| 14. Consistency in Terminology and Instructions | Did RA maintain consistency and standardization in terminology and instructions across all cases? | 0 | 1 | 2 | 3 | 4 | 0 = Completely inconsistent; 1 = Mostly inconsistent; 2 = Partially inconsistent; 3 = Mostly consistent; 4 = Fully consistent and standardized. |
| **Active Control Group (Sham Treatment) Checklist for Intervention Steps**  **Code:** | | | | | | | |
| **Step** | **Description** | **Score** | | | | | **Scoring Explanation** |
|  |  | **0** | **1** | **2** | **3** | **4** |  |
| 1. Reconfirm Eligibility Criteria | Did RA re-evaluate participants based on inclusion criteria to ensure all participants meet the study requirements? | 0 | 1 | 2 | 3 | 4 | 0 = Not confirmed; 1 = Partially confirmed; 2 = Mostly confirmed; 3 = Fully confirmed; 4 = Fully confirmed with no errors. |
| 2. Reconfirm Exclusion Criteria | Did RA re-evaluate participants based on exclusion criteria to ensure no participants meet the exclusion criteria? | 0 | 1 | 2 | 3 | 4 | 0 = Not confirmed; 1 = Partially confirmed; 2 = Mostly confirmed; 3 = Fully confirmed; 4 = Fully confirmed with no errors. |
| 3. Re-explain Study Purpose | Did RA re-explain the study purpose and procedures to participants to ensure they fully understand the significance of their participation? | 0 | 1 | 2 | 3 | 4 | 0 = No explanation; 1 = Incomplete explanation; 2 = Partially explained; 3 = Fully explained; 4 = Fully and clearly explained. |
| 4. Reconfirm Consent Form Completeness | Did RA recheck the completeness of the written informed consent forms for all participants? | 0 | 1 | 2 | 3 | 4 | 0 = Not confirmed; 1 = Partially confirmed; 2 = Mostly confirmed; 3 = Fully confirmed; 4 = Fully confirmed with no errors. |
| 5. Provide eBook Access | Did RA provide the eBook download link via QR code to participants in a timely manner? | 0 | 1 | 2 | 3 | 4 | 0 = Not provided; 1 = Delayed provision; 2 = Partially provided; 3 = Fully provided; 4 = Fully provided without technical issues. |
| 6. Provide eBook Reading Instructions | Did RA provide detailed instructions on how to download and read the eBook, including specific steps and support contacts? | 0 | 1 | 2 | 3 | 4 | 0 = No guidance; 1 = Basic guidance; 2 = Partial guidance; 3 = Full guidance; 4 = Full and specific guidance. |
| 7. Confirm eBook Download and Reading | Did RA confirm participants have successfully downloaded and started reading the eBook, including follow-ups on progress and technical issues? | 0 | 1 | 2 | 3 | 4 | 0 = No confirmation; 1 = Partial confirmation; 2 = Mostly confirmed; 3 = Fully confirmed; 4 = Fully confirmed and all issues resolved. |
| 8. Respond to Participants' Questions | Did RA appropriately address or clarify participants' questions after providing the eBook? | 0 | 1 | 2 | 3 | 4 | 0 = No response; 1 = Partial response; 2 = Mostly responded; 3 = Fully responded; 4 = Full and clear response. |
| 9. Consistency in Terminology and Instructions | Did RA maintain consistency and standardization in terminology and instructions across all cases? | 0 | 1 | 2 | 3 | 4 | 0 = Completely inconsistent; 1 = Mostly inconsistent; 2 = Partially inconsistent; 3 = Mostly consistent; 4 = Fully consistent and standardized. |

| **Supplement 1-3 The Research Assistant Training Manual** | | | |
| --- | --- | --- | --- |
|  |  |  |  |
| **Training Content** | **Training Objectives** | **Training Activities** | **Completed** |
| 1. Understanding Type 1 Diabetes (T1D) and the Needs of Transition Patients | Gain an in-depth understanding of T1D pathophysiology, particularly the unique needs and challenges of transitioning patients. | **Theoretical Learning:** Covers T1D pathophysiology, patient self-management challenges (e.g., insulin dependence, blood glucose fluctuations), and psychosocial impacts. |  |
|  |  | **Simulated Patient Interaction:** Role-play activities simulating communication scenarios with transitioning patients to practice explaining treatment plans, establishing adherence, and addressing patient concerns and resistance. |  |
| 2. Participant Recruitment and Screening | Master the precision of screening and recruiting participants who meet the inclusion and exclusion criteria of the study. | **Case Study and Discussion:** Analyze past recruitment cases to understand challenges and solutions in the recruitment process. |  |
|  |  | **Recruitment Simulation:** Conduct screening simulation using hypothetical participant information, simulating the screening and communication process to strengthen screening judgment skills. |  |
|  |  | **Questionnaire Tool Operation:** Familiarize with the use of screening questionnaires and conduct simulated entry and data management exercises. |  |
| 3. Intervention Group Guidelines | Correctly set up participant codes and accounts, and guide participants in downloading and using the Podcast. | **Set Participant Codes and Accounts:** Conduct practical training on account setup using realistic simulation scenarios to ensure data confidentiality. |  |
|  |  | **Download and Usage Instructions:** Demonstrate how to use QR codes to download the Podcast, explaining the importance of each step. |  |
|  |  | **Guiding Voice Feedback:** Deeply learn PRIDE steps and simulate scenarios for guiding voice feedback, practicing how to handle different types of participant feedback and providing constructive suggestions. |  |
|  |  | **Support and Clarify Technical Issues:** Simulate technical support exercises to ensure each assistant can provide effective support. |  |
|  |  | **Ensure Consistency in Communication:** Use standard communication templates to conduct multiple scenario-based simulations to check for consistency in language and terminology. |  |
| 4. Control Group Guidelines | Guide participants in downloading and using the e-book. | **Provide e-Book Download and Usage Instructions:** Demonstrate how to use QR codes to download the e-book and conduct simulation exercises to ensure all participants can use it correctly. |  |
|  |  | **Support and Clarify Technical Issues:** Simulate technical support exercises to ensure each assistant can provide effective support. |  |
|  |  | **Ensure Consistency in Communication:** Use standard communication templates to conduct multiple scenario-based simulations to check for consistency in language and terminology. |  |
| 5. Treatment Fidelity (TF) | Ensure consistency and standardization of all intervention content and implementation processes. | **Design (Treatment Design):** Familiarize with all details of the intervention design, including the weekly Podcast release, voice feedback arrangements, and control group e-book reading guidance. |  |
|  |  | **Training:** Conduct at least 10 hours of clinical shadowing training, observing interactions between senior clinical staff and patients, documenting case records, and regularly holding review meetings and reflective exercises. |  |
|  |  | **Delivery of Treatment:** Monitor the intervention process, provide on-site guidance, record all sessions, randomly analyze recordings, and ensure all interventions are conducted according to design and standard procedures. |  |
|  |  | **Receipt of Treatment:** Conduct communication skills training to ensure participants understand and accept the intervention content. |  |
|  |  | **Enactment of Treatment:** Conduct regular follow-ups, provide additional support and encouragement to help participants effectively implement what they have learned in their daily lives. |  |
| 6. Compliance and Ethical Considerations | Ensure all research activities comply with ethical standards and Institutional Review Board (IRB) approval. | **Research Compliance:** Learn and understand the ethical guidelines and compliance requirements of the study, conduct scenario-based simulations to ensure all research operations meet IRB approval and standards. |  |
|  |  | **Confidentiality Agreement:** Sign confidentiality agreements, learn how to protect participant personal data, and conduct simulated data protection and management exercises. |  |

| **Supplement 1-4 The Intervention Content Checklist for Participant** | | |
| --- | --- | --- |
| **Code:** |  |  |
| **Intervention Item** | **Description** | **Confirmation (✔)** |
| 1. Introduction to Study and Purpose | Understood the purpose and goals of the study explained by the RA. |  |
| 2. Account Setup | Received a unique participant code and account setup guidance for accessing the Podcast. |  |
| 3. Download Instructions for Podcast | Understood how to use the QR code to download and listen to the Podcast episodes. |  |
| 4. Initial Podcast Episode | Listened to the first episode of the Podcast during the session. |  |
| 5. Voice Feedback Instructions | Understood how to provide voice feedback based on PRIDE steps as explained by the RA. |  |
| 6. Demonstration of Providing Feedback | Observed a demonstration by the RA on how to record and upload voice feedback. |  |
| 7. Technical Support Guidance | Received guidance on resolving common technical issues (e.g., downloading or accessing the Podcast). |  |
| 8. Contact Information for Support | Received contact details for technical support and study-related questions. |  |
| 9. HbA1C Goal Setting | Completed the setting of HbA1C target values as part of the intervention. |  |

**Supplement 2** Detailed Description of Study Scales

| **Scale** | **Description** |
| --- | --- |
| Basic Information Questionnaire | The Basic Information Questionnaire consists of 13 items, developed based on the transition theory framework, with a content validity index of 0.97, confirming its theoretical alignment by expert review. |
| Diabetes Distress Scale (DDS) | This 17-item questionnaire, developed by Polonsky et al. (2005), includes four subscales. For the outcome indicator of “Well-being: Emotional Distress”, three subscales— “emotional burden,” “physician-related distress,” and “regimen-related distress”—will be used, totaling 14 items in the original version and 15 in the Chinese version. Initially scored on a 6-point scale, the Chinese version was adjusted to a 4-point Likert scale to enhance participant usability. Each item is rated from 1 (no distress) to 4 (severely distressed), with higher scores indicating more significant distress.  The original DDS scale demonstrated strong validity, correlating with younger age, higher depression, insulin use, poorer self-care, and elevated lipid levels, and exhibited high reliability with Cronbach’s alpha over 0.93 (Polonsky et al., 2005). In the Chinese version, content validity assessment led to adding “Feeling that diabetes limits my freedom” in the Emotional Burden subscale, resulting in Cronbach’s α values of 0.88–0.90 and a test-retest reliability of 0.81. (Liu et al., 2010). The fourth subscale, “Interpersonal Distress,” will be used to measure “Relationship Comfort: Diabetes-Related Interpersonal Distress” in this study. |
| Diabetes Knowledge Questionnaire-24 (DKQ-24) | This 24-item questionnaire was developed by Garcia et al. (2001), and items are answered “Yes,” “No,” or “I don’t know”; 1 point is awarded for each correct answer. The total score ranges from 0 to 24 points, with higher scores indicating more excellent knowledge of diabetes. The original questionnaire was developed for type 2 diabetes, but items 6 and 13 have been modified for relevance to T1D. Item 6, “If I have diabetes, my children are very likely to have diabetes,” is changed to “false,” and item 13, “Medication therapy controls diabetes better than appropriate diet and exercise,” is changed to “true.” These modifications have been validated in T1D patients (Chiang 2020-2022).  The DKQ-24 showed construct validity, as evidenced by significant score improvements in the intervention group, indicating its sensitivity to educational impact. The Cronbach’s α for the subgroups ranged from 0.73 to 0.84 with an overall value of 0.78 (Garcia et al., 2001). In the Chinese version, age and education were found to significantly predict diabetes knowledge, with a Cronbach’s α of 0.89. (Hu et al., 2013). |
| Diabetes Self-Care Behavior Scale | This 39-item questionnaire was originally developed in Chinese by Wang (2010) and measures self-care behaviors, including “medication management (insulin injection)”, “healthy eating (diet control)”, “blood glucose monitoring (self-monitoring)”, “physical activity (regular exercise)”, “problem-solving”, “risk reduction”, and “healthy coping (psychosocial adaptation and stress management)”. It scored on a 5-point Likert scale. Each item is rated from 1 (not achieved at all) to 5 (completely achieved), with scores ranging from 39 to 195, where higher scores signify superior self-care. The CVI is 0.92, and Cronbach's α is 0.87. |
| Perceived Diabetes Self-Management Scale (PDSM) | This 8-item questionnaire was developed by Wallston et al. (2007). It scored on a 5-point Likert scale ranging from 1 (strongly disagree) to 5 (strongly agree); the total score ranges from 8 to 40, with higher scores indicating higher confidence in diabetes self-management.  The construct validity of PDSM is supported by its positive correlation with self-reported self-care activities and negative correlation with glycemic control measures, and Cronbach’s alpha is .834 (Wallston et al., 2007). The CVI is 0.75, and Cronbach’s α is 0.93 for the Chinese version (Lin et al., 2011). |
| Interpersonal Distress | This 3-item questionnaire is the subscale of the DDS (Polonsky et al., 2005) and will be used to assess the outcome indicator of “Relationship Comfort: Diabetes-Related Interpersonal Distress”. It includes three items: “Feeling that friends or family are not supportive enough of my self-care efforts (e.g., planning activities that conflict with my schedule, encouraging me to eat the “wrong” foods),” “Feeling that friends or family do not appreciate how difficult living with diabetes can be,” and “Feeling that friends or family do not give me the emotional support that I would like.”  Refer to the English and Chinese versions of the DDS questionnaire for reliability, validity, and scoring (Liu et al., 2010; Polonsky et al., 2005). Cronbach’s alpha for this subscale in the Chinese version is 0.88 (Liu et al., 2010). |
| Diabetes Family Conflict Scale-Revised (DFSC-R) | This 19-item questionnaire, developed by Hood et al. (2007), includes two dimensions: “direct management tasks” and “indirect management tasks.” Direct management tasks refer to conflicts directly related to diabetes care, such as: "During the past month, I have argued with my parent(s) about the results of blood sugar monitoring." Indirect management tasks refer to conflicts not directly related to diabetes care, such as: " During the past month, I have argued with my parent(s) about telling friends about diabetes.” Each item is scored on a 3-point Likert scale, with responses ranging from 1 (never argue) to 3 (always argue), resulting in a total score range of 19 to 57, where higher scores indicate greater conflict.  The original DFCS-R scale demonstrated strong concurrent validity, with higher scores correlating with poorer glycemic control and elevated diabetes-related family stress, and exhibited high reliability with Cronbach’s alpha over 0.85 (Hood et al., 2007). |

References of scales

Chiang , Y. T. (2020-2022). *Study on Development and Evaluation of Mobile APP "Health Care CEO" for Patients with Type 1 Diabetics Aged 16-25: Continued Research Project (1) (Chinese)* (MOST 109-2314-B-182-057- MY2) [Grant].

Garcia, A. A., Villagomez, E. T., Brown, S. A., Kouzekanani, K., & Hanis, C. L. (2001). Development of the Spanish-language diabetes knowledge questionnaire. *Diabetes Care*, *24*(1), 16-21. <https://doi.org/10.2337/diacare.24.1.16>

Hood, K. K., Butler, D. A., Anderson, B. J., & Laffel, L. M. (2007). Updated and revised diabetes family conflict scale. *Diabetes Care*, *30*(7), 1764-1769. <https://doi.org/10.2337/dc06-2358>

Hu, J., Gruber, K. J., Liu, H., Zhao, H., & Garcia, A. A. (2013). Diabetes knowledge among old adults with diabetes in Beijing, China. *Journal of Clinical Nursing*, *22*(1-2), 51-60.

Lin, S. M., Lin, S. L., Wu, Y. C., Chang, C. M., & Wu, H. L. (2011). Validity and Reliability of a Chinese Translation of a Perceived Diabetes Self-Management Scale. *Journal of Nursing and Healthcare Research*, *7*(3), 198-206 (Chinese). <https://doi.org/10.6225/JNHR.7.3.198>

Liu, M. Y., Tai, Y. K., Hung, W. W., Hsieh, M. C., & Wang, R. H. (2010). Relationships between emotional distress, empowerment perception and self-care behavior and quality of life in patients with type 2 diabetes. *The Journal of Nursing*, *57*(2), 47-60 (Chinese). <https://doi.org/10.6224/JN.57.2.49>

Polonsky, W. H., Fisher, L., Earles, J., Dudl, R. J., Lees, J., Mullan, J., & Jackson, R. A. (2005). Assessing Psychosocial Distress in Diabetes. *Development of the Diabetes Distress Scale*, *28*(3), 626-631. <https://doi.org/10.2337/diacare.28.3.626>

Wallston, K. A., Rothman, R. L., & Cherrington, A. (2007). Psychometric properties of the perceived diabetes self-management scale (PDSMS). *Journal of Behavioral Medicine*, *30*(5), 395-401. <https://doi.org/10.1007/s10865-007-9110-y>

Wang, H. F. (2010). *Self-care behavior and self-care outcome among children and adolescents with type 1 diabetes mellitus.* [Department of Health Service Administration, China Medical Universty]. Taichung, Taiwan (Chinese).
